# The Effect of Extender Particle Size on the Glass Transition Temperature of Model Epoxy Coatings

**DOI:** 10.3390/polym12010196

**Published:** 2020-01-12

**Authors:** Muhammad Ahsan Bashir, Martin Gjerde Jakobsen, Vivian Beate Farstad

**Affiliations:** Jotun Performance Coatings, Jotun A/S, 3211 Sandefjord, Norway

**Keywords:** inorganic extender, epoxy-amine coating, feldspar, nanosilica, glass transition temperature

## Abstract

Inorganic extenders are important constituents of 2K thermosetting epoxy-amine coatings and their physical properties play an important role in the final properties of the organic coatings. The effects of extender particle size and loading (i.e., the amount of extender in component A or in the total formulation) on the glass transition temperature (*T_g_*) of model epoxy-amine coatings were studied in this work with differential scanning calorimetry (DSC) and dynamic mechanical analysis (DMA). The obtained results show that the particle size and loading of feldspar particles (from 25 wt% to 70 wt%) do not influence the glass transition temperature of the model epoxy-amine coating significantly. In general, the smaller the particle size the lower the glass transition temperature of the coating but this depression in *T_g_* seems negligible when seen relative to the change in extender particle size. Similar observations are reported for two model coatings having the same lamda (*Λ*) value but with silica of very different particle sizes (i.e., nanosilica and micron sized silica).

## 1. Introduction

Among thermosetting polymers, 70% of the market share is captured by epoxy resins (excluding polyurethanes) and 60% of the globally produced epoxy resins is employed in the coatings industry due to their low shrinkage during cure, good adhesion with various substrates, excellent corrosion and chemical resistance, high strength and durability, and good compatibility with numerous commercially important materials. Commercial epoxy coatings can be 1K or 2K (i.e., 1 component or 2 components, respectively, with K used as an abbreviation of the German word for component). 1K epoxy coatings contain anionic or cationic initiators (in one package), which under heat or photoirradiation, can initiate the polymerization of the epoxy monomer. In 2K epoxy coatings, generally, the component containing the epoxy resin (along with other additives like solvent(s), pigment(s), defoaming agent etc.) is termed as the Component A while the second component, termed as Component B, contains the hardener or curing agent. Component A and B are mixed before application. Thermosetting 2K epoxy formulations can be cured by compounds that are reactive towards the epoxy functional group such as, amines, thiols, organic acids, anhydrides, alcohols, and phenols. For liquid epoxy coatings intended to cure at ambient temperatures and below, amine functional curing agents are most widely used. The type of the epoxy resin and curing agent used along with the curing conditions can have a significant effect on the final properties of the coating [1,2].

Commercial epoxy coatings contain additives and fillers which may help in easier processing during production and application, cost reduction, and improving the final properties of the coating (e.g., fracture toughness of the epoxy resin is reported to be increased by using nanosilica [3]). Among fillers, inorganic extenders constitute a major part of the total formulation. These inorganic extenders (sometimes called extender pigments) include kaolin (aluminum silicates), calcium carbonates, talc (magnesium silicate), mica (hydrous aluminum potassium silicates), silica (silicon dioxide), wollastonite (calcium metasilicates), barite (barium sulphate), aluminum oxide etc. Generally, these extenders are inexpensive and therefore, used to reduce the production cost of coatings but their physical and chemical properties (e.g., surface treatment with chemicals like silanes) can impact the final properties of the coating. Surface area, porosity, and particle size distribution (PSD) are among the important physical properties of extenders as they impact the oil absorption of extenders which in turn can influence the processing and final properties of the coatings [4,5].

Most of the research work investigating the role of extender properties in impacting mechanical and thermal properties of cured epoxies is related to nanocomposites. The particle size of the extenders (e.g., silica) becomes important, especially, in the nanometer (nm) range. It is believed that in coatings and nanocomposites an immobilized polymer layer of about 1 nm is formed around the surface of the extender particles. In this layer, known as the interaction region, the conformational entropy and chain kinetics are significantly different from the bulk of the material. For nano-sized extender particles the volume fraction of this interaction region is higher than that for micron-sized particles and, therefore, the properties of coatings and composites containing nanoparticles are different from those materials having their micron-sized analogs as extenders [6,7].

The glass transition temperature (*T_g_*) is an important thermal property of organic coatings which contributes in determining their application area and service life properties. Various researchers have investigated the influence of extender particle size on the *T_g_* of thermosetting epoxies. Sun et al. [7] used diglycidylether of bisphenol-A (DGEBA) resin and hexahydro-4-methylphthalic anhydride (HMPA) hardener along with different particles like silica, silver, aluminum, and carbon black. These particles were of different sizes. The authors showed that at high amounts of silica, silver, and aluminum, the *T_g_* of cured epoxy containing nanoparticles was lower than the *T_g_* of the epoxy containing a similar type of microparticles which was attributed to the increase in free volume due to the enhanced resin-filler interface using nanoparticles. Liu et al. [8] dispersed DGEBA in a commercial product containing pre-dispersed colloidal nanosilica particles (30–31 wt%) in methylisobutylketone (MIBK) and cured the resultant formulations with 4,4- diaminodiphenylmethane (DDM) to obtain silica amounts in the range of 10% to 70%. DSC analysis of the cured samples showed that, in comparison to the neat epoxy sample, the *T_g_* first decreased with up to 30% nanosilica amount (i.e., 40 °C reduction in *T_g_* was noticed) followed by an increase in *T_g_* with up to 50% nanosilica amount. At 70% nanosilica content, no *T_g_* was observed. The authors suggested that at low extender content, *T_g_* decreased due to the plasticization effect of nanosilica whereas at high extender content the motion of the polymer chains was highly hindered by the particles leading to an increase in *T_g_*. According to Chen et al. [9] commercially available nanosilica dispersions contain surface treated nanosilica. These authors used various analytical techniques and estimated that such nanosilica can have an organic layer on the surface of the particles which may account for 3 wt% of the dried powder. Their results showed that good dispersion of nanosilica up to 20 wt% into model epoxy-amine nanocomposite can result in 50 °C reduction in the *T_g_* of the cured model nanocomposite (in comparison to the system without nanosilica). Dittanet et al. [3] showed that silica nanoparticles of sizes from 23 to 170 nm do not impact the *T_g_* of epoxy cured with piperidine by changing the volume concentration of nanoparticles from 0% to 30%. Note that the silica nanoparticles used by these authors were surface treated with organosilane. No other information was provided about the type of organosilane used as it would be important to understand whether the organosilane played a role in the observed *T_g_* independence of silica particle size or not. Similar results were obtained with respect to the *T_g_* of the nanocomposite when glass spheres (average diameter of 42 µm) were combined with silica nanoparticles to simulate a bimodal particle size distribution [10]. When using epoxy resin cured with piperidine and silica nanoparticles of 20 nm and 80 nm average diameter, the results reported by Liang et al. [11] were similar to Dittanet et al. [3]. Preghenella et al. [12] studied the effect of fumed nano-silica amount on the *T_g_* of low molecular weight DGEBA based epoxy, cured with a polyamide curing agent. The amount of silica with the same particle size was varied in the range of 3.3 to 9.2 vol%. The authors showed that the *T_g_* of the cured resin filled with silica is lower in comparison to neat cured epoxy up to a silica content of 6.4 vol% followed by an increase at a silica content of 9.2 vol%. It was proposed by the authors that the initial *T_g_* depression (up to 6.4 vol% of filler content) was probably due to the ability of the silica nanoparticles to prevent a complete cure of the epoxy which lead to a reduction in crosslinking degree. In the case of incomplete reaction during the curing period, DSC heating scans show an exothermic event which can be attributed to the heat released during the reaction of epoxy with the curing agent. No such exothermic events were presented by the authors in DSC heating scans as indication of incomplete cure of the used epoxy resin. At the highest filler content, the *T_g_* was increased due to the increased physical immobilization of polymer chains near the filler surface. Recently, El-Fattah et al. [13] investigated the effect of fumed nanosilica particle size and amount on the *T_g_* of room temperature cured epoxy coating. By using FTIR, the authors showed that silanol groups on the silica surface chemically react with the epoxy groups due to the local heat produced during ultrasonication and that the extent of reaction in smaller particles (average particle size of 7 nm) was higher than that in larger particles (average particle size of 30 nm). However, the effect of silica particle size on the *T_g_* of coatings was not significant as was expected due to this reaction. At similar amounts of silica nanoparticles, the *T_g_* of the coating containing 7 nm diameter particles was found to be 7.5% lower than the *T_g_* of the neat epoxy, whereas, this difference was 4.5% for the coating containing 30 nm diameter particles. Reduction in *T_g_* was noticed up to 2 wt% of both silica types after which the *T_g_* of the coating was found to be similar to the neat epoxy coating. The authors attributed this behavior to poor dispersion at higher nanosilica amounts.

The above-mentioned literature review shows that the effect of extender particle size on the *T_g_* of epoxy nanocomposites is not completely understood in the model systems where mostly one extender was used. Regarding 2K organic epoxy coatings, studies showing the effect of extender particle size on the *T_g_* of the coatings are scarce in the open literature. In addition, several different silica types have been used in these studies. It is well-known that fumed, precipitated, and colloidal silica have very different physical properties (particle size, particle size distribution, surface area, pore volume, pore diameter, pore size distribution) which may lead to different dispersibility characteristics of these silica types [6,14] and, therefore, may have been a reason for the mixed results obtained by different researchers. In this work the aim is to investigate the effect of extender particle size and loading (i.e., the amount of extender in component A or in the total formulation) on the *T_g_* of model epoxy-amine coatings by using commercially important extenders. Furthermore, the effect of extender particle size on the *T_g_* of model systems with the same lamda value (*Λ*) was also studied. *Λ* is the ratio of pigment volume concentration (PVC) to critical pigment volume concentration (CPVC). PVC indicates the volumetric concentration of extenders (or pigments) in the coating. CPVC is representative of the PVC corresponding to the random tightest possible packing of the extender (or pigment) particles and the minimum amount of binder necessary to fill the interstices between extender particles. *Λ* value less than 1 (i.e., PVC <CPVC) typically indicates that the coating film is continuous and contains only polymer (or binder) and pigments. *Λ* values very close to or higher than 1 (i.e., PVC ≥CPVC) indicate that the coating film has air pockets around the extender particles due to lack of polymer. The importance and role of PVC and CPVC in coating properties was reviewed by Perera et al. [4]. Differential Scanning Calorimetery (DSC) and Dynamic Mechanical Analysis (DMA) were mainly used in this study to measure the *T_g_* of the model coatings.

## 2. Materials and Methods

Epoxy resin used in this work was Epokukdo YD-128 obtained from the Kukdo Chemical Co., Ltd., Korea, which is a liquid type standard epoxy resin derived from bisphenol-A with epoxy equivalent weight (EEW) of 187 g per equivalent (g·eq^−1^). Phenalkamine curing agent used to cure the epoxy was obtained from the Cardolite Corporation, USA, with active hydrogen equivalent weight (AHEW) of 162 g·eq^−1^. Extenders used in this study included feldspar (from Sibelco, Norway), calcite (Omya, Malaysia), alumina (Ankerpoort NV, The Netherlands), talc (Imerys, France), Spherilex DP- 0115 amorphous silica (Evonik, Germany), and nanosilica (OOCAP SAS, France). Coatings grade of xylene and n-butanol were used as solvents. Defoaming agent BYK-A 530 was obtained from BYK, Germany.

**Figure 1:**
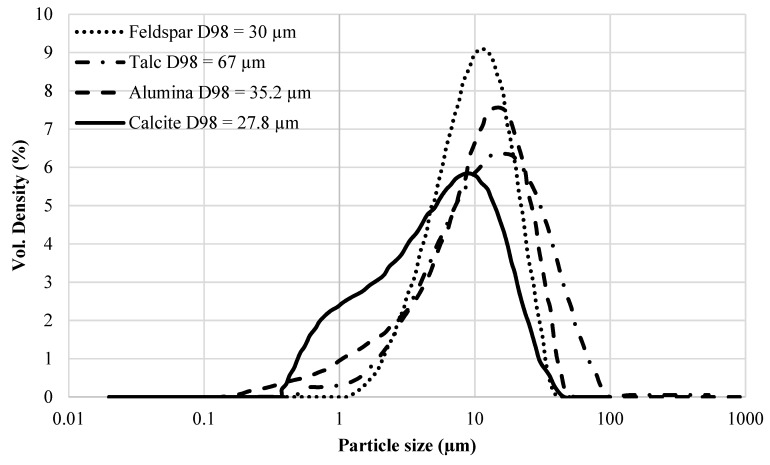
Particle size distribution of different extenders used with D98 mentioned in the legend.

Particle size distribution (PSD) of selected extenders was obtained according to ISO 13320. Samples were collected from thoroughly mixed containers using a spoon. Particle size measurements were obtained on a Mastersizer 3000 instrument using Hydro G dispersion unit with water (23 °C) as dispersing agent. Samples were manually pre-dispersed in a small beaker by using a pipette before being added to the Hydro G. All extenders were pre-dispersed in water except talc which was pre- dispersed in 70% EtOH. Samples were added to Hydro G until 5–15% obscuration was reached. During measurement 20% ultrasound was applied continuously and the stirrer/pump speed was set to 2400 rpm. The samples were analyzed by manual mode and measurement duration set to 10 s. Consecutive measurements were made until stable results were achieved. The PSDs of different extenders are shown in Figure 1, whereas Table 1 shows the respective values for D_50_ and D_98_ for each extender.

Differential Scanning Calorimetry (DSC) analysis of the samples was performed on a Q200 DSC instrument from TA Instruments. 5 to 10 mg of sample was placed in a hermetic aluminum DSC cell. A hole was made in the lid of the DSC cell prior to running DSC analysis in order to avoid effects of evaporating species on the *T_g_* of the samples. All samples were analyzed by conventional heat/cool/heat cycle with heating and cooling rates of 10 °C·min^−1^ under continuous nitrogen flow (i.e., each sample was first heated from −50 °C to 150 °C at a heating rate of 10 °C·min^−1^ (referred to as the first DSC heat scan in this work), then cooled down to −50 °C at a cooling rate of 10 °C·min^−1^, followed by a heating scan −50 °C to 150 °C at a heating rate of 10 °C·min^−1^ (referred to as the second DSC heat scan in this work). The *T_g_* of the samples was estimated from the heating scans as the mid- point of the glass transition region by using Universal Analysis software from TA instruments.

DMA analysis was performed on a Q800 DMA model from TA Instruments with film tension clam and DMA Multi-Frequency—Strain mode. For all samples, oscillation amplitude and force track were kept at 5.0 µm and 125.0%, respectively. Each sample film was first cooled down to −50 °C followed by heating up to 150 °C or 200 °C at a constant heating rate of 4 °C·min^−1^. The obtained data was analyzed by using Universal Analysis software from TA instruments.

For morphology of the extenders used, a Hitachi SU3500 Scanning electron microscope was used.

Table 2 shows the model formulation used for preparing component A in a speed mixer at 2000 rpm. All components A were mixed with the curing agent on a stoichiometric basis in a speed mixer and drawdowns with wet film thickness (WFT) of 400 µm were prepared on a mylar sheet. The curing cycle consisted of 1 day at room temperature and six days at 60 °C.

## 3. Results and Discussion

**Figure 2:**
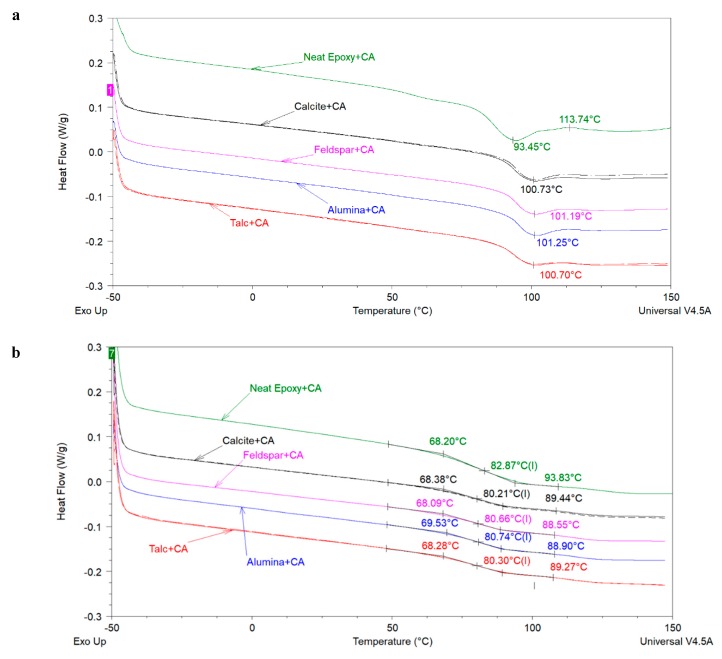
First heating (**a**) and Second heating scan (**b**) of the coatings prepared with extenders of different particles sizes. For coatings with calcite and talc, 2 samples were analyzed.

Figure 2 compares the first and second heating scans of cured epoxy samples without and with different extenders. In these formulations, extender loading was kept constant at 45 wt% in component A. A small exothermic event was noticed in the first heating curve of neat epoxy centered at 113.7 °C indicating negligible residual reactivity. In all other samples with extenders no such exothermic event was noticed indicating fully cured systems. The first heating scan (see Figure 2a) shows that the glass transition temperature (*T_g_*) region of the coating with neat epoxy is different from that of the epoxy coatings filled with extenders. This is shown by using the peak endothermic temperature (*T_p_*) which is 93.45 °C for the neat epoxy sample whereas for coatings filled with extenders *T_p_* is close to 101 °C and shows some dependence on particle size of the used extenders. Overall, the first DSC heating scans suggest that addition of extenders increases the *T_g_* of the epoxy coatings in comparison to the epoxy coating without any extender and that the *T_g_* shows almost no dependence on the particle size of the used extender (see Table 3). This increase in *T_g_* upon addition of extenders may be attributed to the fact that at high loadings, extenders can hinder the polymer chain mobility in cured epoxy-amine systems [8]. It is important to mention that the same region of the first DSC heating curves of cured epoxies (in conventional DSC) is known to provide information about the enthalpy relaxation, therefore, one may also suggest that the presence of extenders probably leads to higher enthalpy relaxation rates providing higher *T_g_* of the filled systems as compared to the neat epoxy system but evaluating the effect of extender particle size on enthalpy relaxation is beyond the scope of this work. Figure 2b and Table 3 show that the *T_g_* values estimated from the second DSC heating curve of the epoxy coating with and without extenders were very similar. In fact, the *T_g_* of the neat epoxy coating was found to be slightly higher than the coatings with extenders. Once again, no effect of extender particle size was seen on the *T_g_* (estimated from the second DSC heating scan) of filled coatings.

**Figure 3:**
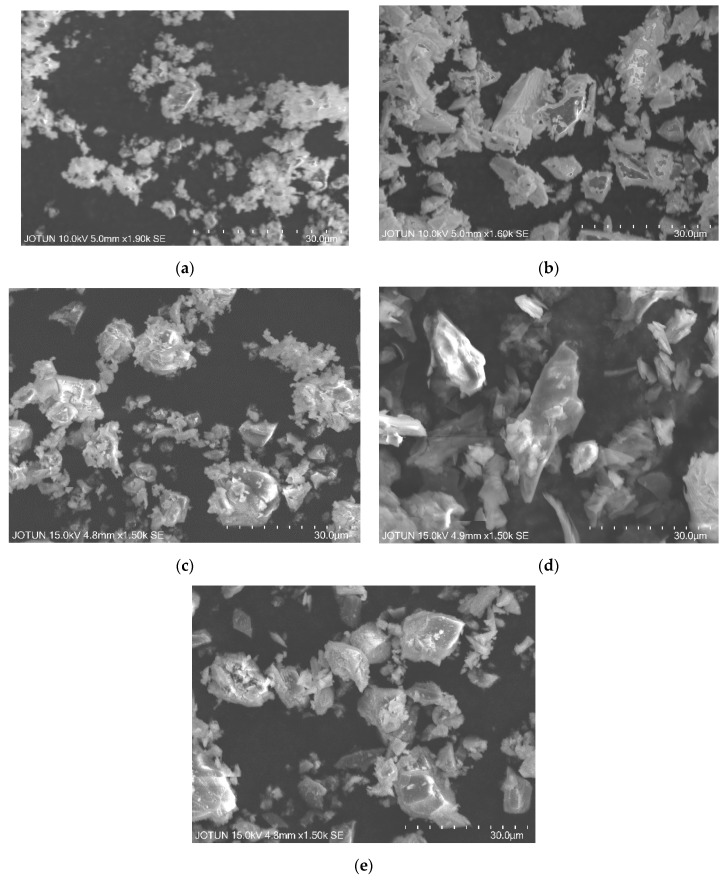
SEM micrographs showing morphology of different extenders. (**a**) Calcite, (**b**) Feldspar, D_98_ = 30 µm, (**c**) Alumina, (**d**) Talc and (**e**) Feldspar, D_98_ = 100 µm.

**Figure 4:**
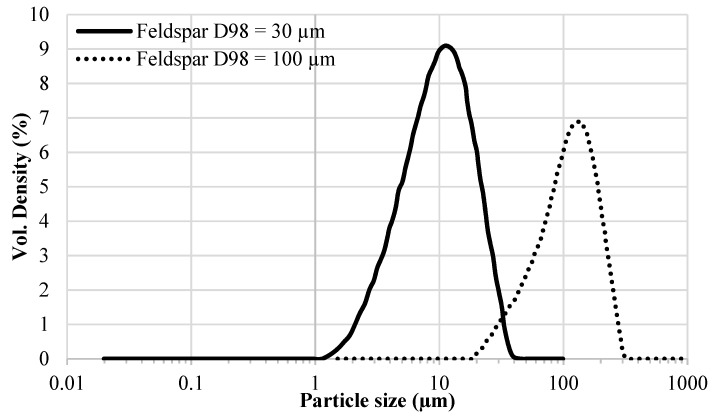
Particle size distribution of two Feldspar grades.

With respect to the effect of the particle size of extenders on the *T_g_* of epoxy-amine coatings, it should be noted that while the morphology of the used extenders is different (see Figure 3) their PSDs are not very different as shown in Figure 1. Except calcite, all other extenders have a similar shape and breadth of PSD. In addition, these extenders are chemically different from each other. Therefore, two feldspar grades with considerably different PSDs (see Figure 4) but similar morphology (compare Figure 3b and Figure 3e) were used to further investigate the effect of extender particle size on the *T_g_* of the epoxy-amine model coatings at different extender loadings (i.e., 25 wt%, 45 wt%, and 70 wt% in component A). Two grades of the same extender also ensured that the extender chemistry has been kept very similar. All other parameters (mixing, WFT, curing agent, and curing conditions) were kept as for the samples mentioned above.

**Figure 5:**
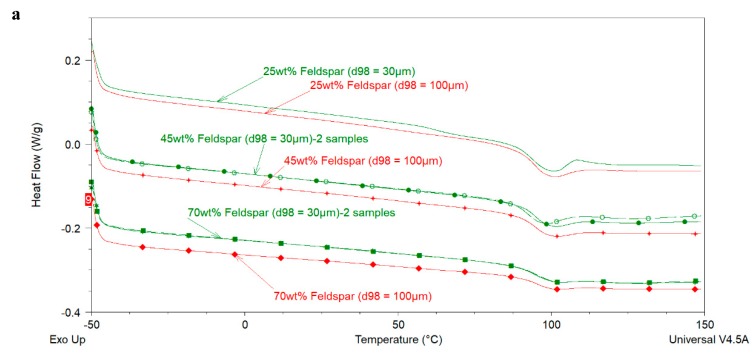

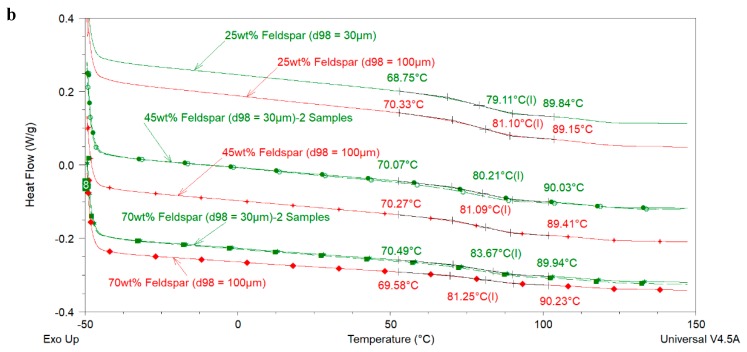
First heating (**a**) and Second heating scan (**b**) of the coatings prepared with feldspars of different particle sizes.

Figure 5 shows the effect of extender loading and particle size on the 1st and 2nd heating DSC curves of epoxy-amine coatings whereas Table 4 presents the corresponding values of *T_g_* for each sample shown in Figure 5a,b. The 1st heating curves indicate that none of these samples showed residual reactivity and with increasing extender loading or particle size there is no significant effect on the glass transition region of these coatings. *T_g_* values estimated from 1st and 2nd heating DSC curves (see Table 4) do not show significant dependence both on the extender loading and particle size, especially, on considering the fact that the particle loading and size were varied about three times. These results are in agreement with the work of Sun et al. [7] who used micrometer and nanometer sized silica particles and showed that with up to 40 wt% loading of micron sized silica particles, the *T_g_* of the epoxy cured with anhydride remains unchanged (in comparison to neat cured epoxy). However, with nanometer sized silica, the *T_g_* of the cured epoxy decreased at higher loadings (i.e., >10 wt%).

**Figure 6:**
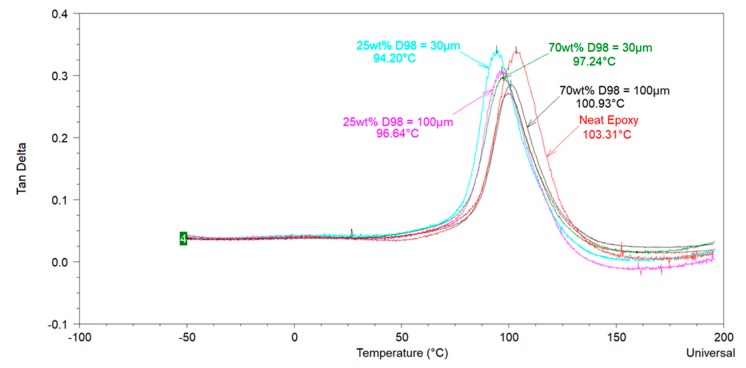
Tan Delta curves of epoxy coatings with different sized feldspars obtained from DMA.

Figure 6 shows the *tan δ* curves of the selected samples with different extender loadings and particle sizes. Observations from DMA analysis are similar to those made from the DSC analysis i.e., (i) extenders decrease the *T_g_* of the cured epoxy-amine system, and (ii) a slight effect of particle size on the *T_g_* of the cured system can be noticed and one might conclude that at the same extender loading (or pigment volume concentration (PVC)) the *T_g_* of the coating with smaller extender particles is lower than that of the coating with larger particles. However, considering the 3-fold change in particle size and loading this change in *T_g_* of the coating is probably insignificant. Nevertheless, it is important to mention that long term service life properties of epoxy coatings with extenders of different particle sizes might be different as the physical and chemical ageing behavior of these coatings may be affected by such differences in the physical properties of the extenders.

**Figure 7:**
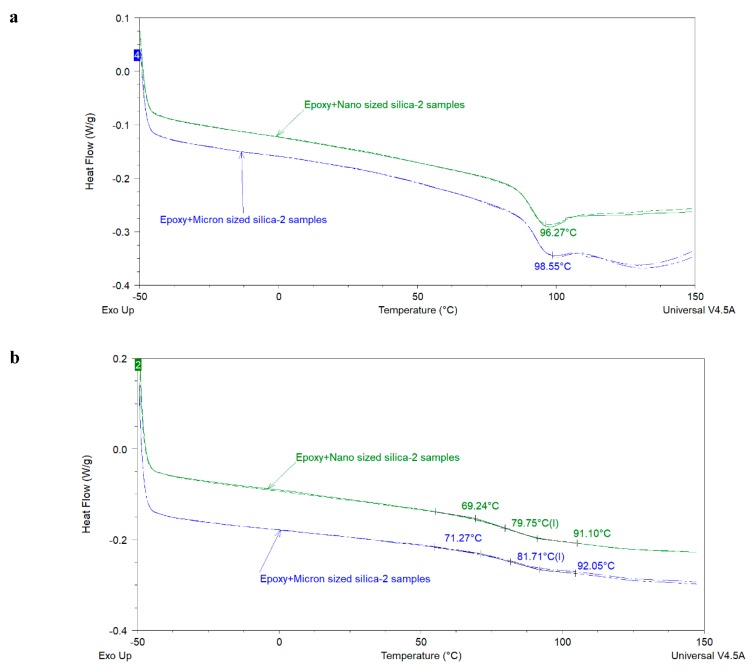
First heating (**a**) and Second heating scan (**b**) of epoxy coatings prepared with nanosilica and micrometer sized silica at the same *Λ* value of 0.51.

**Figure 8:**
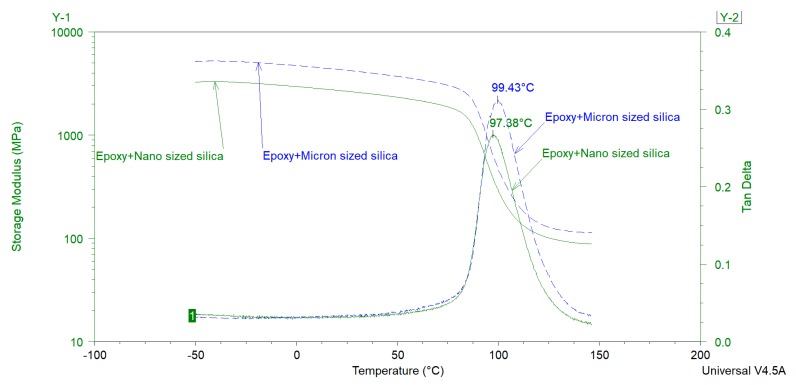
DMA analysis of the epoxy coatings with different silica particle sizes but same *Λ* value of 0.51.

In order to further investigate the effect of particle size on *T_g_* of epoxy-amine coatings, two more formulations were prepared where the nanosilica and micronsized silica were used as extenders but lamda (*Λ*) of the final coating (i.e., component A + component B) was kept constant at 0.51. For these coatings, all other components (mentioned in Table 2), curing agent, WFT, and curing conditions were kept similar to the previously discussed samples. Average particle sizes of nano- and micron- sized silica were 80 nm and 13.5 µm, respectively. Oil absorption of the nanosilica was 168 g of oil per 100 g of extender whereas that of micron-sized silica was 37 g of oil per 100 g of extender reflecting significant differences in their surface areas. DSC and DMA analysis of these samples, shown in Figure 7 and Figure 8, respectively (and Table 5), confirms the above-mentioned results. i.e., the smaller sized extenders decrease the *T_g_* of the epoxy coatings in comparison to their larger counterparts at the same *Λ*, most probably due to the increased resin-filler interface [7] but this depression in *T_g_* is insignificant when compared with the extent of variation in the particle size of extenders. However, it is important to mention that these results are based upon a model coating where one extender type was used at a time. In commercial 2K epoxy-amine coatings generally multiple extenders of different sizes are used. Therefore, more detailed and systematic studies are needed in order to understand the effect of extender particle size on the *T_g_* of the coatings. Furthermore, it is important to mention that enthalpy relaxation effect(s) and extender(s) particle size effect(s) on the *T_g_* of the coatings need to be separated.

## 4. Conclusions

The effect of the particle size of extenders on the glass transition temperature (*T_g_*) of model epoxy-amine coatings was investigated by selecting extenders of commercial importance with different particle sizes and particle size distributions. DSC and DMA analysis of the cured epoxy- amine systems showed that the particle size of the extenders (e.g., feldspar and silica used in this work) slightly impacts the *T_g_* of the cured epoxy-amine coatings i.e., the smaller the size of the extender particles the lower the *T_g_* of the final cured coating. However, the extent of *T_g_* depression is not proportional to the extent of change in extender particle size i.e., a three-fold reduction in average particle size of the extenders used, reduced the *T_g_* of the coating (obtained from the first heating curve of the DSC) by a maximum of 3 °C (at the same PVC). Therefore, it can be concluded that changes in average particle size of the epoxy-amine coatings do not have a significant influence on the *T_g_* of the cured coating. Increasing the extender amount up to 70 wt% also showed no significant influence on the *T_g_* of the coating.

## Figures and Tables

**Table 1:** D_50_ and D_98_ of the extenders used in this work.

Extender	D_50_ (µm)	D_98_ (µm)
Calcite	5.7	28
Feldspar	6.8	30
Alumina	8.0	35
Talc	12.9	67

**Table 2:** Example of formulation for component A.

Description	% Weight
Epoxy Resin 828	38.4
Xylene	9.0
N-Butanol	7.0
Soya Lecitin 100%	0.4
Extender	45.0
BYK-A 530	0.3
**Total**	**100.0**

**Table 3:** *T_g_* of the coatings estimated from 1st and 2nd DSC heating curves.

System	D_98_ of Extender (µm)	*T_g_* 1st Heating (°C)	*T_g_* 2nd Heating (°C)
Neat Epoxy	-	86.6	82.9
Epoxy + Calcite	28	94.9	80.2
Epoxy + Feldspar	30	95.1	80.7
Epoxy + Alumina	35	94.8	80.7
Epoxy + Talc	67	93.9	80.3

**Table 4:** *T_g_* of the coatings estimated from 1st and 2nd DSC heating curves for samples with different loadings of feldspars with different particle sizes. PVC = pigment volume concentration based upon total formulation. * = average of two values.

wt%	PVC (%)	*T_g_* 1st Heating	*T_g_* 2nd Heating	*T_g_* 1st Heating	*T_g_* 2nd Heating
0	0	86.6	82.9	86.6	82.9
		**D_98_ = 30 µm**		**D_98_ = 100 µm**	
25	20	91.8	79.6	95.1	81.2
45	34	93.9 *	77.6 *	94.9	81.0
70	61	94.7 *	83.5 *	95.9	81.3

**Table 5:** *T_g_* of the coatings estimated from 1st and 2nd DSC heating curves for samples with nanosilica and micrometer sized silica. N/A = not applicable.

*Λ*	*T_g_* 1st Heating	*T_g_* 2nd Heating	*T_g_* 1st Heating	*T_g_* 2nd Heating
N/A	86.6	82.9	86.6	82.9
	**Nanosilica**		**Micrometer Silica**	
0.51	90.9	79.7	92.9	82.9
